# Reduced frequency and functional defects of CD4^+^CD25^high^CD127^low/−^ regulatory T cells in patients with unexplained recurrent spontaneous abortion

**DOI:** 10.1186/s12958-020-00619-7

**Published:** 2020-06-10

**Authors:** Li Luo, Xun Zeng, Zhongying Huang, Shan Luo, Lang Qin, Shangwei Li

**Affiliations:** 1 Department of Obstetrics and Gynecology, West China Second University Hospital, Sichuan University, Chengdu, China; 2 Key Laboratory of Birth Defects and Related Diseases of Women and Children, Sichuan University, Ministry of Education, Chengdu, China

**Keywords:** CD4^+^CD25^high^CD127^low/−^ regulatory T cell, Forkhead box transcription factor P3, Unexplained recurrent spontaneous abortion, Tolerance

## Abstract

**Background:**

Unexplained recurrent spontaneous abortion (URSA) is defined as two or more consecutive pregnancy losses, generally of unknown cause; it is related to a failure of fetal–maternal immunological tolerance. Regulatory T cells (Tregs) exert immunosuppressive effects, which are essential to maintain fetal–maternal immunological tolerance and regulate immune balance. In this study, we used the specific cell-surface phenotype of CD4^+^CD25^high^CD127^low/−^ Tregs to investigate the number and suppressive function of Tregs isolated from the peripheral blood of patients with URSA with the aim of expanding our understanding of their role in URSA.

**Methods:**

We isolated a relatively pure population of peripheral CD4^+^CD25^high^CD127^low/−^ Tregs and CD4^+^CD25^−^ responder T cells (Tresps) from the patients with URSA and normal fertile nonpregnant control women via fluorescence-activated cell sorting. We compared the frequency, suppressive capacity, and forkhead box transcription factor P3 (FOXP3) expression of Tregs in the peripheral blood between patients with URSA and normal controls.

**Results:**

The frequency of CD4^+^CD25^high^CD127^low/−^ Tregs in the peripheral blood was lower in URSA patients than in the controls (*P* < 0.05). The mean fluorescence intensity of FOXP3 and *FOXP3* mRNA expression in Tregs was also significantly lower in the URSA patients (*P* < 0.01). Tregs suppressed the activity of autologous Tresps stimulated with anti-CD3/CD28 beads in a concentration-dependent manner, with the strongest suppression occurring in cocultures with a 1:1 Treg:Tresp ratio in both groups; however, patient-derived Tregs exhibited a poorer capacity to suppress the proliferation of autologous Tresps than the Tregs from normal controls (*P* < 0.01). Moreover, Tregs isolated from URSA patients inhibited the proliferation of Tresps from normal controls less potently than the Tregs from normal controls (*P* < 0.01), and Tresps from URSA patients were less effectively suppressed by autologous Tregs than by those from normal controls (*P* < 0.01). Tresp activity were intact in both groups.

**Conclusions:**

We observed a lower frequency of peripheral CD4^+^CD25^high^CD127^low/−^ Tregs with lower FOXP3 expression in the peripheral blood of URSA patients. In addition, highly purified Tregs from patients with URSA exhibited impaired suppressive effects. The defect in immune regulation in URSA patients appears to be primarily related to impaired Tregs, and not to increased resistance of Tresps to suppression. Our findings reveal a potential novel therapeutic target for URSA.

## Background

Recurrent spontaneous abortion (RSA), defined as 2 or more consecutive pregnancy losses, affects approximately 1–2% of prospective mothers [1, 2]. The etiology of RSA is multifactorial; it can be related to fetal chromosomal factors, anatomical abnormalities, and hormonal problems. However, in more than half of RSA cases, the precise cause remains unknown; this phenomenon is termed unexplained RSA (URSA) [3, 4]. Recent evidence suggests that URSA is related to a failure of fetal–maternal immunological tolerance [5].

Successful pregnancy requires various immunological adaptations to facilitate attachment, implantation, placentation, and fetal–maternal tolerance [6]. Disturbances of these adaptations are related to pregnancy complications, such as RSA and preeclampsia [7–9]. Dynamic immunological changes throughout normal conception include downregulation of T helper (Th) 1 and Th17 responses, general Th2-skewing of T lymphocyte responses, and enrichment of uterine natural killer cells and regulatory T cells (Tregs) in the first and second trimesters of pregnancy [7, 10, 11]. Uterine natural killer cells represent approximately 70% of all uterine lymphocytes; they are abundant in the decidua during the commencement of implantation, and regulate decidual blood vessel remodeling, decidual cell proliferation, and trophoblast invasion [12–14]. T lymphocyte proportions in the uterus are more variable; they comprise approximately 10 to 20% of decidual leukocytes in the first trimester [15], and their proportion increases over the course of pregnancy [16]. CD25^high^FOXP3^+^ Tregs with immunosuppressive properties, which account for 5% of CD4^+^ T cells, are important to sustain a successful pregnancy [17]. Treg expansion can be detected in the follicular phase of the menstrual cycle, and the serum estradiol-driven increase peaks at ovulation [18]. The proportions of both peripheral and decidual Tregs continue to increase in early pregnancy, and reach their peaks at mid-gestation, after which they gradually decline until 6–8 weeks after delivery to basal levels [17, 19, 20]. Tregs exert several biological effects during pregnancy; they induce decidual support for embryo implantation through contact with other immune cells [8], develop the tolerance towards paternal antigens [19, 21], and promote proper maternal vascular remodeling for robust placental development [22]. Although some studies have demonstrated that lower than average proportions and disrupted functions of Tregs are closely related to the occurrence of URSA [23–26], their findings remain inconclusive due to the limitations of their approaches for identifying Tregs.

Previous studies have typically used the CD4^+^CD25^+^ surface phenotype to investigate the involvement of Tregs in pregnancy loss [27–30]. However, CD25 is not a unique Treg marker as it is also expressed on activated T cells [31, 32]. Thus, previous findings may have been confounded by the presence of contaminating non-Tregs that express CD25. Only cells that express the highest levels of CD25 (CD25^high^) exert a potent immunosuppressive ability [32, 33].

Forkhead box transcription factor P3 (FOXP3)—the main transcriptional regulator for the induction, development, and suppressive ability of Tregs—is unique and specific to Tregs [31, 34–37]. Dysregulated FOXP3 expression in Tregs is associated with autoimmune diseases, and may induce the failure of immune tolerance in a dose-dependent manner [38]. Upregulation of FOXP3 expression induces the conversion of naïve T cells into Tregs [39], whereas a reduction in FOXP3 expression is related to immunosuppressive Treg dysfunction [40, 41]. Unfortunately, as FOXP3 is an intracellular protein, this marker cannot be used to isolate Tregs and is of limited value in Treg functional studies.

Recent studies have demonstrated that surface expression of the IL-7 receptor (CD127) inversely correlates with FOXP3 expression, such that CD4^+^CD127^low/−^ cells largely overlap with FOXP3^+^ cells; thus, CD127 can be used as a cell surface marker to distinguish Tregs from activated CD4^+^ T cells [42, 43]. Activated effector T cells that express high levels of CD127 and do not mediate immunosuppressive functions are also present within the CD4^+^CD25^high^ T cell population [43, 44]. Therefore, instead of the CD4^+^CD25^+^CD127^low/−^ phenotype for Tregs used in previous studies [25], in this study, we relied on high expression of CD25 and low or no expression of CD127 to strictly distinguish Tregs (CD4^+^CD25^high^CD127^low/−^) from activated T cells and investigate the number and suppressive capacity of Tregs isolated from the peripheral blood of patients with URSA with the aim of expanding our understanding of their role in URSA.

## Methods

### Study subjects

All the URSA patients screened in our study had experienced at least 2 consecutive abortions during the first trimester and had no successful pregnancy record. They all underwent a series of investigations to exclude known risk factors for RSA. The diagnosis of URSA was made according to the following criteria to exclude any verifiable causes. First, pelvic examination and transvaginal ultrasound were performed to confirm an anatomically normal uterus. If abnormalities were suspected, hysterosalpingography or diagnostic hysteroscopy was conducted for further confirmation. Second, normal karyotypes for both partners and the absence of embryonic chromosomal abnormalities were confirmed. Third, the cervical mucus was cultured to rule out chlamydia and ureaplasma infections. Fourth, endocrine (luteal function defect, polycystic ovarian syndrome, hyperandrogenemia, and hyperprolactinemia) and metabolic (insulin resistance, diabetes, hyperthyroidism, and hypothyroidism) diseases were excluded. Fifth, factor V Leiden and prothrombin gene mutations, and protein C, protein S, and antithrombin III activity were measured to rule out inherited thrombophilia. Finally, autoantibodies, including anticardiolipin antibodies and antinuclear antibodies, were analyzed to exclude autoimmune diseases. In addition, the partners of all RSA patients were tested for normal semen status.

All control subjects were normal fertile women who had at least 1 successful naturally conceived full-term pregnancy and had no history of spontaneous abortion, stillbirth, preterm labor, ectopic pregnancy, preeclampsia, or otherwise abnormal pregnancies. The URSA patients and normal fertile control subjects enrolled in our study were not taking oral contraceptives, and all were nonpregnant and had regular menstrual cycles (26–30 days). Peripheral blood samples were obtained from all participants at least 3 months after their last abortion or pregnancy in the mid-luteal phase (on day 7 after the urinary luteinizing hormone surge).

Of 113 women with a history of RSA screened for participation in the study, 60 (27 with 2 consecutive abortions, 20 with 3, 8 with 4, 4 with 5, and 1 with 6) were enrolled (mean age: 31.7 ± 5.1 years, range: 22–40 years); 53 were excluded because of abnormalities of the uterus (*n* = 8), endocrine disease (*n* = 13), genetic abnormalities (n = 13), immunological disorders (*n* = 7), thrombotic disease (*n* = 10), or partners with semen abnormalities (*n* = 2). Sixty fertile women (mean age: 30.9 ± 5.6 years, range: 23–41 years) were enrolled as controls. There was no significant difference in age between both groups.

Patients were recruited from the reproductive center at the West China Second University Hospital. The protocol was approved by the Ethics Committee of West China Second University Hospital. Prior to being enrolled in the study, all subjects provided written informed consent to participate, along with additional informed consent for blood sample collection.

### Collection of peripheral blood mononuclear cells

Peripheral blood mononuclear cells (PBMCs) were isolated from heparinized peripheral blood samples by density-gradient centrifugation with Ficoll-Hypaque (MD Pacific, Tianjin, China). The viability of all isolated PBMCs was > 90%, as assessed by the Trypan blue exclusion assay. The PBMCs were cultured in RPMI 1640 (Sigma, St. Louis, MO, USA) supplemented with 10% fetal bovine serum, 2 mM l-glutamine, 100 units/mL penicillin, and 100 μg/mL streptomycin (all from Gibco BRL, Grand Island, NY, USA).

### Antibodies

Phycoerythrin-labeled anti-CD25, Alexa Fluor® 647-labeled anti-CD127, PerCP-Cy5.5-labeled anti-CD4, Alexa Fluor® 488-labeled FOXP3, and mouse isotype controls were all purchased from BD Biosciences (San Diego, CA, USA). T cells were stimulated with anti-CD3/28 beads (DYNAL Life Technologies, Paisley, UK).

### Flow cytometry

T lymphocytes were incubated with the fluorescently labeled antibodies in the dark at 4 °C for 20–30 min. Then, the cells were washed with phosphate-buffered saline containing 2% bovine serum albumin (Merck, Darmstadt, Germany). Cell surface marker analysis was performed using a FACScan™ flow cytometer (Becton Dickinson, San Diego, CA, USA). The cells were stained with different combinations of the indicated monoclonal antibodies and with Alexa Fluor® 488-labeled anti-FOXP3 after fixation and permeabilization according to the manufacturer’s instructions. Appropriate isotype controls were used in all staining procedures. In all experiments, gates were set based on negative isotype controls.

### Sorting/isolation of T cell subsets

**Fig. 1:**
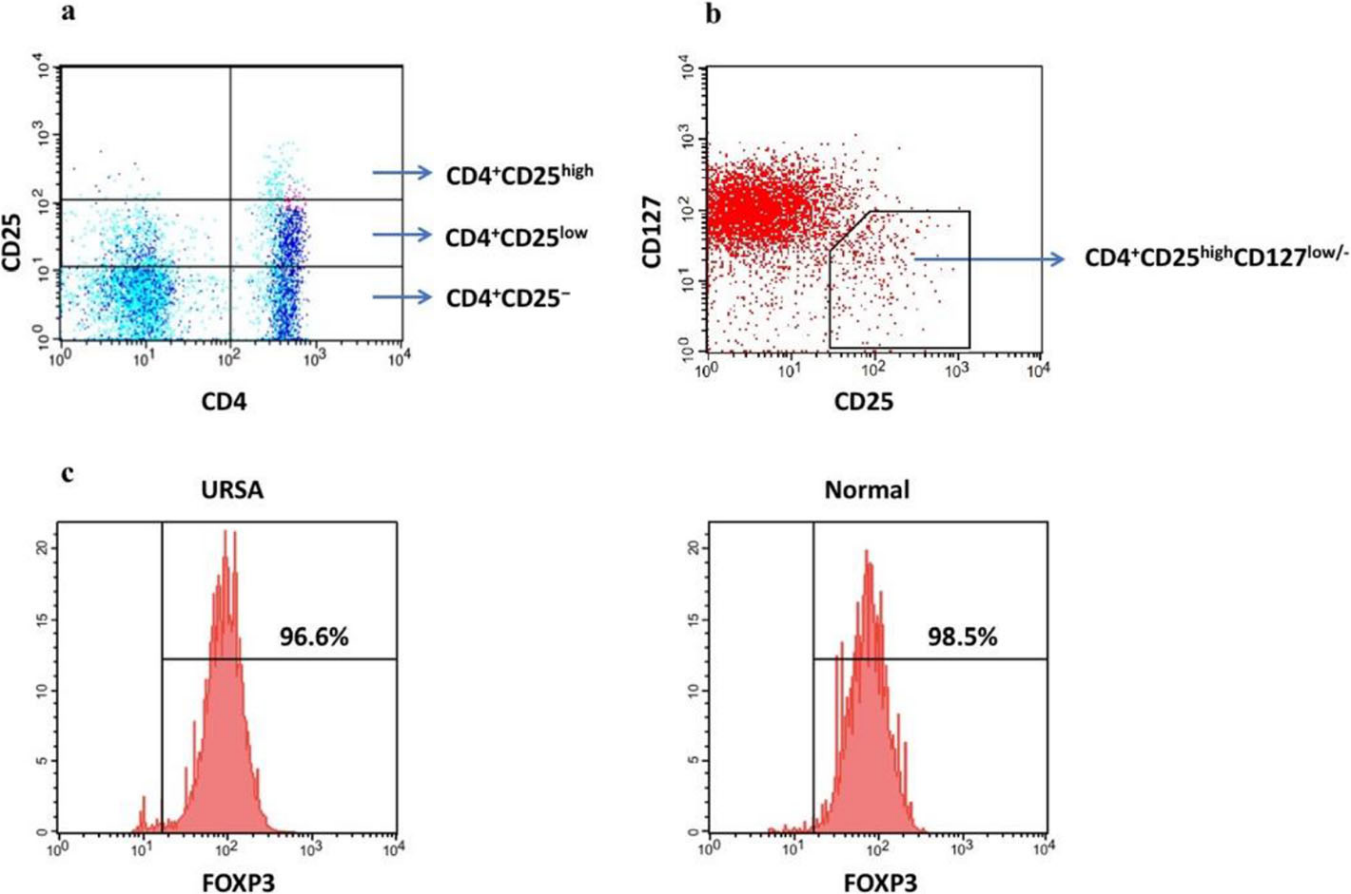
Identifying Tregs and Tresps and analysis of FOXP3 expression in isolated CD4^+^CD25^high^CD127^low/−^ T cells. We immunolabeled PBMCs with PerCP-Cy5.5-labeled anti-CD4, phycoerythrin-labeled anti-CD25, and Alexa Fluor® 647-labeled anti-CD127. **a** CD4^+^ T cells were gated as CD25^high^, CD25^low^, and CD25^−^ based on the level of CD25 expression in CD4^−^ T cells. CD4^+^CD25^−^ cells were isolated as Tresps. **b** We identified Tregs as CD25^high^CD127^low/−^ cells among the CD4^+^ T cell population. **c** To verify the purity of the sorted Tregs, we stained the cells with Alexa Fluor® 488-labeled anti-FOXP3. The percentage of FOXP3 expression in freshly isolated CD4^+^CD25^high^CD127^low/−^ T cells is shown. The data revealed that sorted Tregs were > 95% positive for FOXP3 in URSA patients (*n* = 12) and normal controls (n = 12). Representative data of a patient with URSA (left panel) and a normal control subject right panel) are shown

Labeled CD4^+^ T cells were sorted using a MoFlo instrument (Beckman Coulter, Brea, CA, USA). The gates for the sorted T cell subsets were set to include only those with CD4-specific fluorescence. The CD4-gated cells were further sorted based on CD25 and CD127 expression (CD4^+^CD25^high^CD127^low/−^ Tregs and CD4^+^CD25^−^ responder T cells (Tresps) (Fig. 1). The purity of the isolated CD4^+^CD25^high^CD127^low/−^ Tregs and CD4^+^CD25^−^ Tresps was > 95%. The viability of T cells from both URSA patients and control subjects was > 95%. The sorted cells were collected into 100% human AB serum (Sigma-Aldrich, Poole, UK) and resuspended in RPMI 1640 medium containing 10% human AB serum, 100 IU/mL penicillin, and 100 μg/mL streptomycin. Isotype controls were used to determine the gating parameters.

### Quantitative reverse-transcription PCR

Total RNA was isolated from sorted CD4^+^CD25^high^CD127^low/−^ Tregs using.

TRIzol™ (Invitrogen, Carlsbad, CA, USA), and was reverse-transcribed into cDNA using a PrimeScript™ RT Reagent Kit (TaKaRa Bio, Shiga, Japan), according to the manufacturer’s instructions. PCRs were run using SYBR® Premix Ex Taq™ II (TaKaRa Bio, Shiga, Japan) in an iCycler iQ™5 Real-Time Detection System (Bio-Rad, Hercules, CA, USA). The following primers were used: GAPDH: 5′-CTGGGCTACACTGAGCACCA-3′ (forward) and 5′-TGAGGTCCACCACCCTGTTG-3′ (reverse), and FOXP3: 5′-AGGTGGCAGGATGGTTTCT-3′ (forward) and 5′-AACAGCACATTCCCAGAGTTC-3′ (reverse). Target mRNA expression was normalized to the level of *GAPDH* mRNA. Relative transcript levels were determined using the 2^−ΔΔCt^ method [45].

### Suppressor assays

The suppressive activity of sorted Tregs on Tresps was evaluated in coculture carboxyfluorescein diacetate succinimidyl ester (CFSE) assays, as described by Venken et al. [22]. Tresps were incubated with 5 μM CFSE (Invitrogen, Karlsruhe, Germany). The labeled and washed Tresps (5 × 10^4^ cells/well) were cultured in duplicate in 96-well round-bottom plates in the presence or absence of sorted Tregs at Tresp:Treg ratios of 0:1, 1:0, 1:1, 2:1, and 4:1 in the presence of anti-CD3/CD28 beads (bead:cell ratio of 3:1; Invitrogen, Karlsruhe, Germany). We selected the 1:1 cell ratio to measure Treg suppressive capacity based on preliminary results. Purified Tresps cultured in the absence of anti-CD3/CD28 beads were used to assess background proliferation. After 5 days of coculture, the cells were harvested and the proliferation of the CFSE-labeled Tresps was analyzed by flow cytometry. All CFSE data were analyzed using Cell-Quest™ (Becton Dickinson, San Jose, CA, USA) and ModFit LT™ V2.0 software (Verity Software House, Topsham, ME, USA). The percentage of suppression was calculated as follows: suppression (%) = 100–100 × (Tresp proliferation in the presence of Tregs/Tresp proliferation in the absence of Tregs).

In separate experiments, anti-CD3/CD28-stimulated CD4^+^CD25^−^ Tresps from normal controls were cocultured with either autologous CD4^+^CD25^high^CD127^low/−^ Tregs or with Tregs from URSA patients at a 1:1 ratio, and vice versa. Suppression of Tresp proliferation by Tregs was evaluated as described above.

### Statistical analysis

Data are expressed as the means ± standard errors of the means. Means were compared using Student’s *t*-test and one-way analysis of variance for data with a normal distribution. Pairwise comparison of multiple groups was conducted with the Student–Newman–Keuls q-test or Games-Howell test. For data not showing a normal distribution, we used the Mann–Whitney U-test and Kruskal–Wallis test. Data analyses were performed using SPSS version 20.0 software. Figures were generated using GraphPad Prism 5.0 software. For all tests, *P* < 0.05 was considered significant.

## Results

### Isolation of CD4^+^CD25^high^CD127^low/−^ Tregs

We used three-color fluorescence-activated cell sorting to isolate CD4^+^CD25^−^ and CD4^+^CD25^high^CD127^low/−^ T cells from normal controls (*n* = 12) and URSA patients (n = 12) (Fig. 1a and b). Phenotypic analysis of the sorted CD4^+^CD25^high^CD127^low/−^ T cells revealed that they comprised mainly FOXP3^+^ cells in both URSA patients and normal controls (96.8 ± 1.7% vs. 97.2 ± 1.9%, *P* > 0.05; Fig. 1c). Thus, the CD4^+^CD25^high^CD127^low/−^ cells displayed a Treg phenotype and were used in the suppressive assays.

### Frequency of Tregs in the peripheral blood of URSA patients

**Fig. 2:**
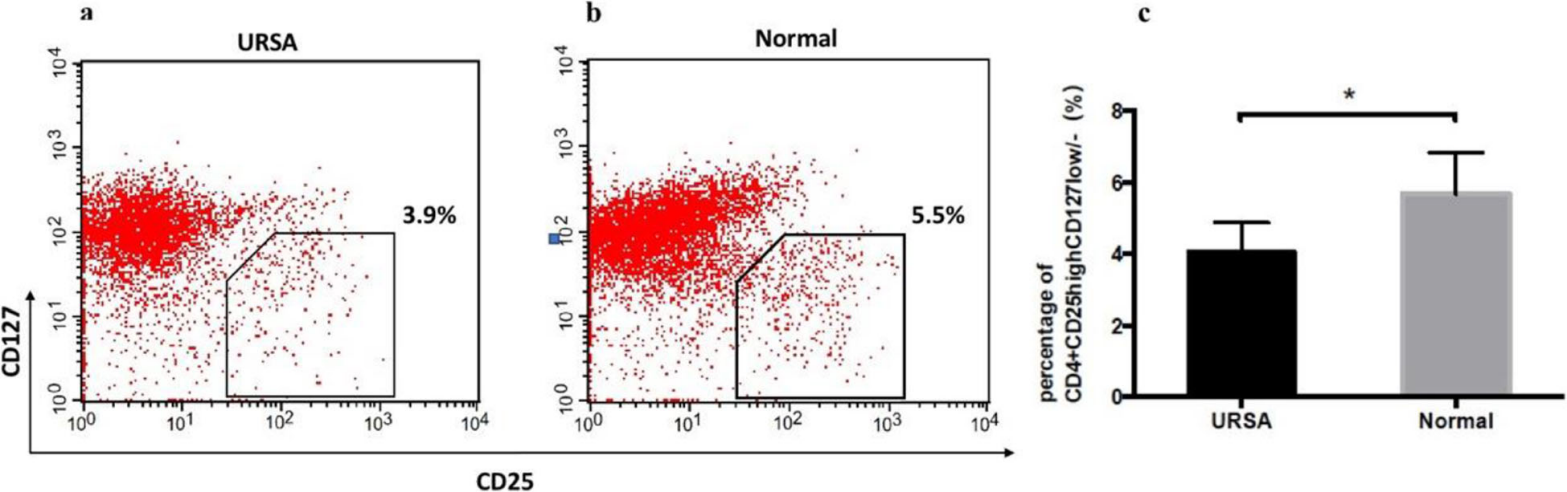
Percentages of CD4^+^CD25^high^CD127^low/−^ Tregs among CD4^+^ T cells. Percentages of CD4^+^CD25^high^CD127^low/−^ Tregs among CD4^+^ T cells in the peripheral blood of URSA patients (*n* = 60) and normal controls (n = 60) are shown. Representative flow cytometry plots for CD4^+^CD25^high^CD127^low/−^ Tregs from (**a**) a patient with URSA and (**b**) a normal control subject. **c** Comparative analysis of the frequencies of CD4^+^CD25^high^CD127^low/−^ Tregs among CD4^+^ T cells in the peripheral blood of URSA patients and normal control subjects (*P < 0.05)

The frequency of CD4^+^CD25^high^CD127^low/−^ Tregs among CD4^+^ T cells in the peripheral blood was significantly lower in URSA patients (*n* = 60) than in the normal controls (n = 60) (4.06 ± 0.35% vs. 5.64 ± 0.49%, *P* < 0.05; Fig. 2).

### FOXP3 expression in peripheral blood CD4^+^CD25^high^CD127^low/−^ Tregs of URSA patients

**Fig. 3:**
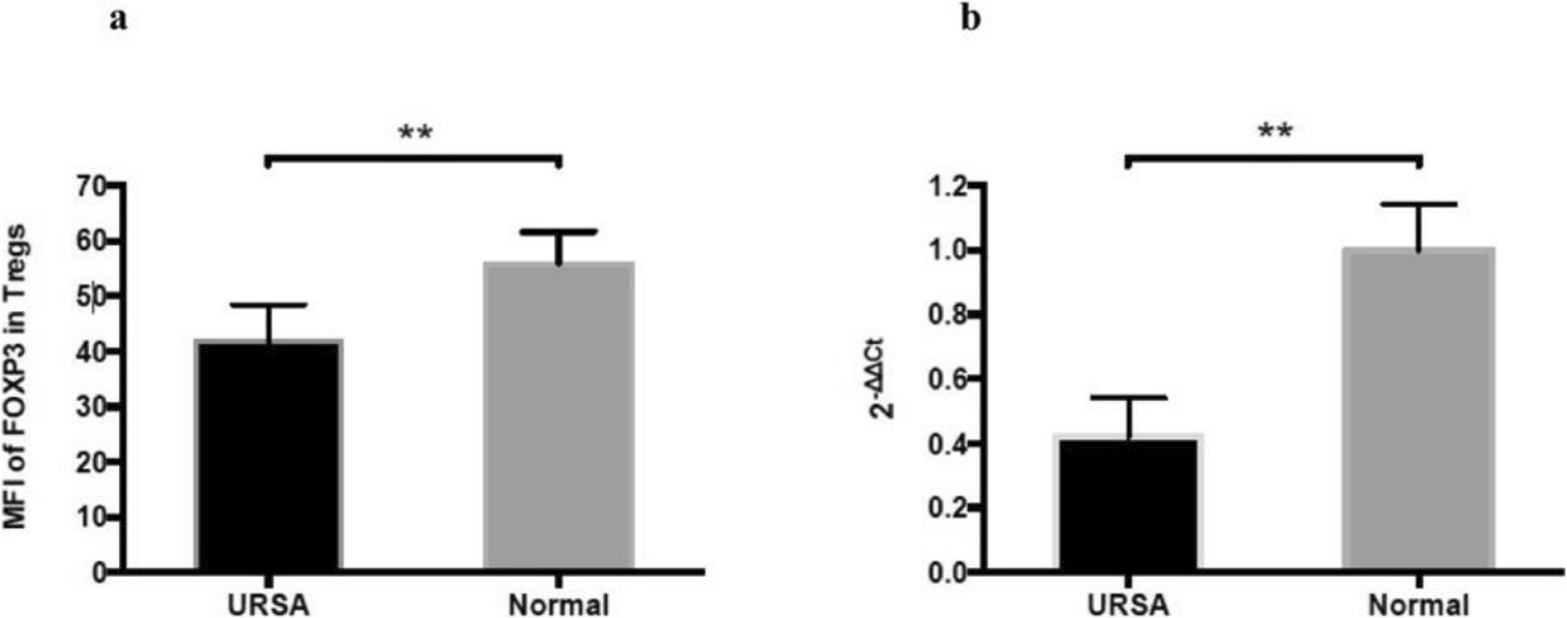
FOXP3 protein and mRNA expression differs between URSA patients and normal control subjects. **a** Comparative analysis of the mean fluorescence intensity of FOXP3 expression in peripheral CD4^+^CD25^high^CD127^low/−^ Tregs in normal controls (n = 12) and URSA patients (n = 12) (**P < 0.01). **b** Comparative analysis of *FOXP3* mRNA expression in peripheral CD4^+^CD25^high^CD127^low/−^ Tregs from normal control subjects (n = 12) and URSA patients (n = 12) (**P < 0.01) by quantitative reverse-transcription PCR. Relative expression was calculated using the 2^−ΔΔCT^ method

The mean fluorescence intensity of FOXP3 was significantly lower in CD4^+^CD25^high^CD127^low/−^ Tregs isolated from URSA patients (*n* = 12) than in those isolated from the normal controls (n = 12) (41.35 ± 5.12 vs. 54.26 ± 4.69, *P* < 0.05; Fig. 3a). In addition, *FOXP3* mRNA levels were significantly lower in CD4^+^CD25^high^CD127^low/−^ Tregs from URSA patients (n = 12) than in those from the controls (n = 12) (*P* < 0.05; Fig. 3b).

### Suppressive function of peripheral CD4^+^CD25^high^CD127^low/−^ Tregs from URSA patients

Sorted CD4^+^CD25^high^CD127^low/−^ Tregs from URSA patients (*n* = 10) and normal controls (n = 10) were used in functional suppressor assays to determine their regulatory potential. CD4^+^CD25^high^CD127^low/−^ Tregs isolated from URSA patients and normal controls were hyporesponsive to anti-CD3/CD28 bead stimulation (2.3 ± 0.3% versus 1.9 ± 0.4%, *P* > 0.05), indicating that CD4^+^CD25^high^CD127^low/−^ T cells from URSA patients exhibited appropriate anergy.

**Fig. 4:**
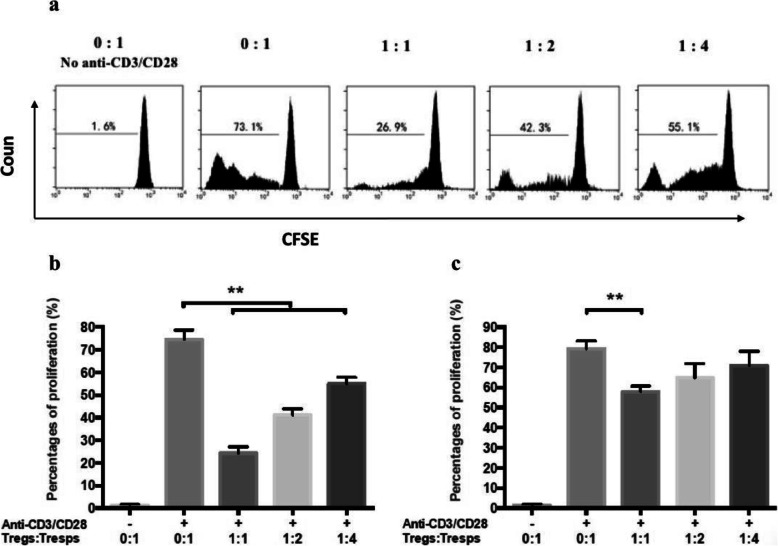
Anti-CD3/CD28-stimulated CD4^+^CD25^−^ Tresp proliferation in the presence of CD4^+^CD25^high^CD127^low/−^ Tregs. **a** Representative flow plot showing the proliferation of Tresps isolated from a normal control. **b** Mean percentage of anti-CD3/CD28 bead-stimulated CD4^+^CD25^−^ Tresp proliferation upon coculture with CD4^+^CD25^high^CD127^low/−^ Tregs at the indicated cell ratios. All cells were isolated from the controls (*n* = 10) (**P < 0.01). **c** Mean percentage of anti-CD3/CD28 bead-stimulated CD4^+^CD25^−^ Tresp proliferation upon coculture with CD4^+^CD25^high^CD127^low/−^ Tregs at the indicated cell ratios. All cells were isolated from patients with URSA (n = 10) (**P < 0.01)

To assess their regulatory properties, we cultured CD4^+^CD25^high^CD127^low/−^ Tregs from normal controls (Fig. 4a and b) and URSA patients (Fig. 4c) in the presence or absence of CD4^+^CD25^−^ Tresps (at Treg:Tresp ratios of 0:1, 1:1, 2:1, and 4:1) stimulated with anti-CD3/CD28 beads. Tregs from both URSA patients (*n* = 10) and normal controls (n = 10) significantly suppressed the proliferation of their respective autologous Tresps at various ratios. The suppressive effect was dose-dependent, and the lowest Tresp proliferation rate was achieved at a 1:1 ratio (24.3 ± 4.9% for control Tresps vs. 58.1 ± 5.6% for URSA patient Tresps). Therefore, further experiments were performed using a 1:1 Treg:Tresp ratio. The proliferation of Tresps from URSA patients did not significantly differ from that of Tresps from the control subjects (75.8 ± 6.5% vs. 80.1 ± 8.2%, *P* > 0.05).

**Fig. 5:**
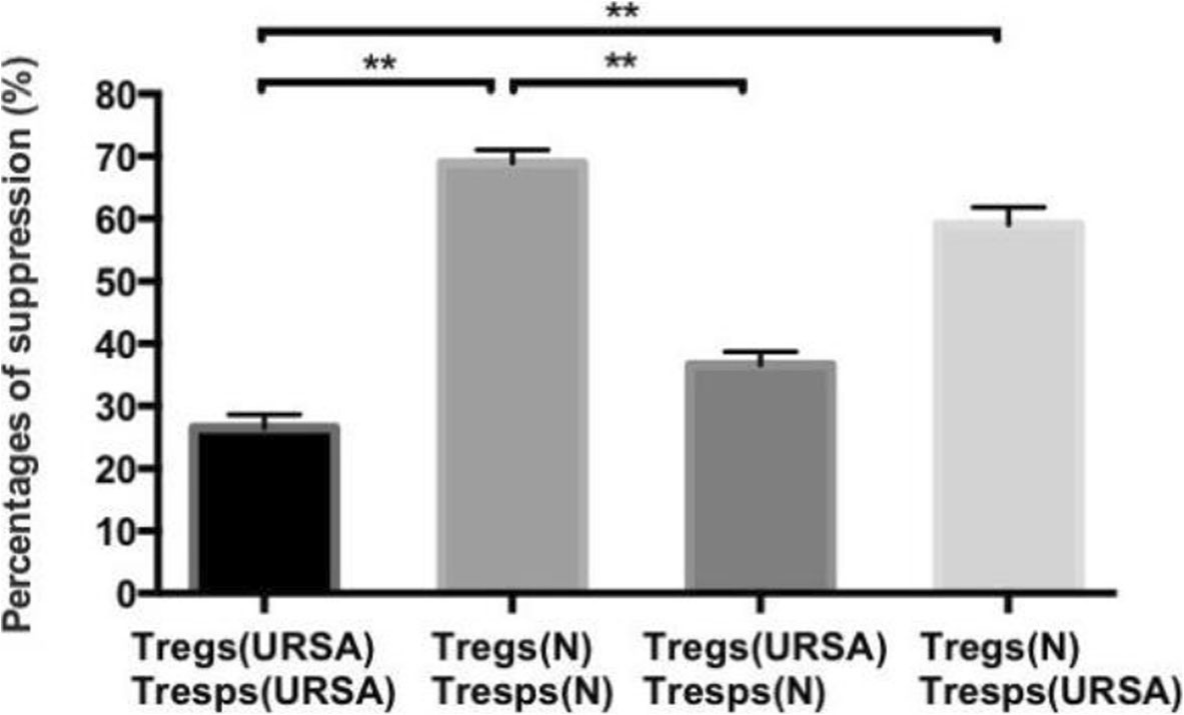
CD4^+^CD25^high^CD127^low/−^ Treg-mediated suppression of CD4^+^CD25^−^ Tresp proliferation. CD4^+^CD25^high^CD127^low/−^ Tregs from URSA patients (n = 10) and normal control subjects (n = 10) were cocultured with autologous or allogeneic anti-CD3/CD28 bead-stimulated Tresps at a 1:1 cell ratio (**P < 0.01)

It was critical to explore whether the loss of Tresp regulation in URSA patients compared to normal controls was due to a defect in CD4^+^CD25^high^CD127^low/−^ Treg function or due to greater resistance of activated CD4^+^CD25^−^ Tresps to suppression. To this end, we cocultured CD4^+^CD25^high^CD127^low/−^ Tregs from the URSA patients (*n* = 10) and controls (n = 10) with autologous or allogeneic Tresps at a 1:1 cell ratio. Peripheral CD4^+^CD25^high^CD127^low/−^ Tregs isolated from URSA patients showed poor activity in suppressing the proliferation of autologous Tresps than Tregs isolated from normal controls (*P* < 0.01; Fig. 5). Additionally, Tresps from normal controls were more effectively inhibited by autologous Tregs than by Tregs from URSA patients (68.9 ± 2.8% vs. 36.6 ± 3.2% suppression, *P* < 0.01; Fig. 5). Similarly, Tresps from URSA patients were inhibited more potently by Tregs from normal controls than by autologous (58.9 ± 4.8% vs. 26.9 ± 3.1% suppression, *P* < 0.01). Tregs from normal controls suppressed the proliferation of autologous Tresps and Tresps from URSA patients to similar levels (*P* > 0.05). Together, these results indicated that the primary regulatory defect in URSA patients is in the function of their peripheral blood CD4^+^CD25^high^CD127^low/−^ Tregs.

## Discussion

URSA has been suggested to be largely associated with a failure of fetal–maternal immunological tolerance, in which Tregs play a crucial role [46]. Evidence suggests that URSA is associated with reduced Treg numbers or function. In early studies, the CD4^+^CD25^+^ phenotype was used to identify Tregs, and later studies refined the phenotype to CD25^high^ or FOXP3^+^ CD4^+^ T cells to more specifically identify Tregs [18, 24]. However, as FOXP3 is a nuclear protein, it cannot be used to isolate Tregs for functional assays. Several studies have shown that FOXP3 expression inversely correlates with CD127 expression, indicating that CD4^+^CD25^+^ T cells with low or no CD127 expression mainly comprise FOXP3-expressing cells [42, 43].

To optimize the isolation of cells that actually function as Tregs, we utilized the specific CD4^+^CD25^high^CD127^low/−^ phenotype to isolate Tregs with the aim of expanding our understanding of their role in URSA. We confirmed that CD4^+^CD25^high^CD127^low/−^ T cells are a highly purified population of FOXP3^+^ Tregs, which allowed us to examine the frequency and function of Tregs in the circulation of URSA patients. URSA patients had a lower proportion of CD4^+^CD25^high^CD127^low/−^ Tregs among the CD4^+^ T cells than normal controls Furthermore, we observed lower expression of FOXP3 on a per-Treg basis at the protein and mRNA levels in URSA patients, which is consistent with the lower frequency of circulating Tregs. These findings are in agreement with reports of lower-than-normal levels of Tregs in patients with URSA [18, 23, 24, 47]. Yang et al. [23] reported that the proportions of CD4^+^CD25^bright^ T cells in both the peripheral blood and decidua of URSA patients were lower than those in normal pregnant control subjects. Wang et al. [47] observed that the frequencies of CD4^+^CD25^+^CD127^low/−^ T cells in the peripheral blood and decidua of URSA patients were lower than those in normal women in early pregnancy. In addition, Mei et al. [24] found that the percentage of CD4^+^CD25^high^ Tregs in the peripheral blood of nonpregnant URSA patients was lower than that in URSA patients who had early miscarriages and normal nonpregnant women. In the decidua, they observed a lower frequency of CD4^+^CD25^high^ Tregs and lower FOXP3 expression in Tregs in URSA patients with early miscarriages than in normal women in early pregnancy.

A few studies have described the peripheral and decidual expansion of CD4^+^CD25^+^FOXP3^+^ Treg populations in women during the first 2 trimesters of pregnancy [48, 49]. Qian et al. [50] reported that the proportion of peripheral CD4^+^CD25^+^CD127^low^ Tregs was not higher in pregnant women with a history of URSA than in nonpregnant women with URSA, whereas the percentage of Tregs, the expression level of CTLA-4 in CD4^+^CD25^+^CD127^low^ cells, and the proportion of CD4^+^FOXP3^+^ cells in the decidua were significantly lower in patients with URSA than in normal controls. On the other hand, Quan et al. [51] reported no differences in the percentages of peripheral CD4^+^CD25^+^ cells among total CD4^+^ cells or FOXP3^+^ cells among CD4^+^CD25^+^ cells between nonpregnant URSA patients, early-term URSA patients, normal women who underwent planned abortions, and normal nonpregnant controls. These studies used different antibody panels to identify Tregs at various phases of the menstrual cycle [18]; both these factors are likely to have affected the proportions of Tregs observed.

Our study is the first to report the isolation of CD4^+^CD25^high^CD127^low/−^ Tregs and CD4^+^CD25^−^ Tresps from the peripheral blood of URSA patients to conclusively determine the localization of the T cell-inhibitory defect. We found that Tregs isolated from patients with URSA suppressed autologous Tresps stimulated with anti-CD3/CD28 beads in a concentration-dependent manner, with the greatest suppression occurring in coculture at a 1:1 Treg:Tresp ratio; however, patient-derived Tregs had poorer suppressive capacity than Tregs from normal controls. Anergy, a hallmark of CD4^+^CD25^high^CD127^low/−^ Tregs, was evident in Tregs from both URSA patients and normal fertile women. In addition, Tresps from URSA patients and control subjects had similar proliferation rates. This result corroborated that the defects in the suppressive activity of Tregs from URSA patients were not due to fluctuations in CD4^+^CD25^−^ Tresp proliferation. Other groups have assessed the function of Tregs in the context of URSA [18, 25]. Arruvito et al. [18] found that RSA patients showed similarly lower proportions of CD4^+^CD25^+^, CD4^+^CD25^high^, and FOXP3^+^ cells within the CD4^+^ population in both the follicular and luteal phases, comparable to the proportions in postmenopausal women. Unlike fertile controls, RSA patients did not exhibit fluctuations in these cell populations during their menstrual cycles. Moreover, the authors found that CD4^+^CD25^+^ cells—deemed Tregs in this study—from RSA patients had lower suppressive activity than those from fertile controls. Bao et al. [25] reported that CD4^+^CD25^+^CD127^dim/−^ Tregs isolated from the decidua of URSA patients had lower suppressive potency than those from normal pregnant controls. They also found that only 2 and 1% of Tregs from URSA patients expressed IL-10 and TGF-β, respectively, compared with 9 and 14%, respectively, in Tregs from gestational controls, and the immunosuppressive function of the Tregs could be partially blocked with neutralizing anti-IL-10 and anti-TGF-β antibodies. However, they did not explore whether the functional T cell suppression deficit was specific to Tregs or intrinsic to Tresps.

We analyzed whether the apparent functional impairment of Tresp suppression observed in URSA patients was partly due to resistance of the patients’ Tresps to suppression. We found that Tregs from patients with URSA were less potent in suppressing the proliferation of Tresps from normal fertile women than Tregs from the normal controls. Tregs from URSA patients also showed less inhibitory activity in cocultures with autologous Tresps than Tregs from the normal controls. Taken together, our experiments demonstrated, for the first time, that the failure of Tresp suppression in URSA patients is predominantly due to an intrinsic Treg defect, rather than the resistance of Tresps to suppression by functional CD4^+^CD25^high^CD127^low/−^ Tregs.

From a clinical perspective, our findings have 2 important implications. First, the phenotype CD4^+^CD25^high^CD127^low/−^ can be used for Treg selection and purification, particularly for clinical immunotherapy applications. Second, CD4^+^CD25^high^CD127^low/−^ Tregs may contribute to the immunopathogenesis of URSA and may be an attractive target for the prevention and treatment of URSA. Strategies to boost Treg abundance and suppressive function for achieving successful gestation in patients with URSA should be evaluated.

## Conclusion

We demonstrated that the CD4^+^CD25^high^CD127^low/−^ phenotype is highly specific for the isolation and purification of Tregs. In addition, we demonstrated deficiencies in peripheral CD4^+^CD25^high^CD127^low/−^ Treg numbers, suppressive function, and FOXP3 expression in patients with URSA. The impaired immunoregulation in patients with URSA is primarily localized to Tregs, not Tresps. Further studies are needed to identify the specific Treg defects in patients with URSA and the mechanisms underlying Treg dysfunction.

## Abbreviations

CFSE: Carboxyfluorescein diacetate succinimidyl ester
FOXP3: Forkhead box transcription factor P3
PBMCs: Peripheral blood mononuclear cells
RSA: Recurrent spontaneous abortion
Th: T helper
Treg: Regulatory T cells
Tresp: Responders T cells
URSA: Unexplained recurrent spontaneous abortion
